# Characterization of circRNA–miRNA–mRNA networks regulating oxygen utilization in type II alveolar epithelial cells of Tibetan pigs

**DOI:** 10.3389/fmolb.2022.854250

**Published:** 2022-09-21

**Authors:** Yanan Yang, Yongqing Li, Haonan Yuan, Xuanbo Liu, Yue Ren, Caixia Gao, Ting Jiao, Yuan Cai, Shengguo Zhao

**Affiliations:** ^1^ College of Animal Science and Technology, Gansu Agricultural University, Lanzhou, China; ^2^ Xinjiang Academy of Animal Sciences, Ürümqi, Xinjiang, China; ^3^ Academy of Agriculture and Animal Husbandry Sciences, Institute of Animal Husbandry and Veterinary Medicine, Lhasa, China; ^4^ State Key Laboratory of Veterinary Biotechnology, Harbin Veterinary Research Institute, Chinese Academy of Agricultural Sciences, Harbin, China; ^5^ College of Grassland Science, Gansu Agricultural University, Lanzhou, China

**Keywords:** noncoding RNA, ATII cells, ceRNA, Tibetan pigs, cellular processes

## Abstract

Understanding the signaling pathway regulatory mechanisms in type II alveolar epithelial (ATII) cells, the progenitor cells responsible for proliferating and regenerating type I alveolar epithelial (ATI) and ATII cells, in Tibetan pigs is beneficial for exploring methods of preventing and repairing cellular damage during hypoxia. We simulated a hypoxic environment (2% O_2_) for culture ATII cells of Tibetan pigs and Landrace pigs, with cells cultured under normoxic conditions (21% O_2_) as a control group, and performed integrated analysis of circular RNA (circRNA)–microRNA (miRNA)–messenger RNA (mRNA) regulatory axes by whole-transcriptome sequencing. Functional enrichment analysis indicated that the source genes of the differential expressed circRNAs (DEcircRNAs) were primarily involved in cell proliferation, cellular processes, and cell killing. A series of DEcircRNAs were derived from inhibitors of apoptosis proteins and led to a key autonomous effect as modulators of cell repair in Tibetan pigs under hypoxia. The significant higher expression of *COL5A1* in TL groups may inhibited apoptosis of ATII cells in Tibetan pigs under lower oxygen concentration, and may lead their better survive in the hypoxia environment. In addition, a competing endogenous RNA (ceRNA) network of functional interactions was constructed that included novel_circ_000898-ssc-miR-199a-5p-*CAV*1 and novel_circ_000898-ssc-miR-378-*BMP*2, based on the node genes ssc-miR-199a-5p and ssc-miR-378, which may regulate multiple miRNAs and mRNAs that mediate endoplasmic reticulum (ER) stress-induced apoptosis and inflammation and attenuate hypoxia-induced injury in ATII cells under hypoxic conditions. These results broaden our knowledge of circRNAs, miRNAs, and mRNAs associated with hypoxia and provide new insights into the hypoxic response of ATII cells in Tibetan pigs.

## Introduction

The Tibetan pig is a unique domestic highland breed in China and has well adapted to the hypoxic environment than other pigs, as the native breed live at Qinghai-Tibet Plateau; indeed, the nucleotide diversity in most regions of the mitogenome is greater in wild Tibetan pigs than in domestic pigs (Li et al., 2013; Li et al., 2016; Wu et al., 2020). Specific characteristics of Tibetan pigs, including their well-developed hearts and lungs, denser pulmonary artery networks, and miRNA–mRNA coexpression regulatory networks in the lungs, facilitate more effective oxygen transport in hypoxic environments (Yang et al., 2021a; Yang et al., 2021b). The lung is the primary respiratory organ that exchanges oxygen and has a large and vascularized epithelial surface area, which is susceptible to hypoxia-induced injury as a “rate-limiting” organ under severely hypoxic conditions (Gallagher and Hackett, 2004; Herriges and Morrisey, 2014). ATI cells and ATII cells were covered the surface of alveolar, and gas exchange relies on the integrity of the epithelium and its dynamic interaction with other cells (Aspal and Zemans, 2020). The hypoxic response of cells depends on regulatory networks at the transcriptional and post-transcriptional levels that result in variations in gene expression (Jozefczuk et al., 2010; Richter et al., 2010). Therefore, cells must be able to sense, respond, and adapt rapidly to short- or long-term hypoxic conditions for optimum survival. Cell turnover is slower in the lungs than in other organs, such as the skin and intestine (Sender and Milo, 2021), and hypoxia (Greco et al., 2019) or hypoxia-induced (Qin et al., 2018) acute lung diseases or injury, including pulmonary fibrosis (Darby and Hewitson, 2016). ATII cells functioning as progenitor cells in the alveoli can regenerate and proliferate into ATII cells or undergo repair and differentiate into ATI cells after physiological insult under hypoxia. The existing literature shows that hepatocyte growth factor (Ito et al., 2014), keratinocyte growth factor (Schmeckebier et al., 2013), and the Wnt/β-catenin (Aspal and Zemans, 2020) signaling pathway can promote the proliferation of ATII cells. In addition, the levels of genes and proteins involved in HIF-related pathways and inflammation activation were found to be significantly increased in ATII cells under hypoxic conditions (Grek et al., 2011; Sherman et al., 2018). Oxidative stress may be the primary reason for ATII cell apoptosis, which could undergo cell death and replaced by myofibroblasts in hypoxia-induced IPF to prevent repair and renewal of the alveolar wall (Dias-Freitas et al., 2016; Alvarez-Palomo et al., 2020). To date, an approach utilizing integrated analysis of protein-coding messenger RNAs (mRNAs) and many noncoding RNAs, i.e., circular RNAs (circRNAs) and microRNAs (miRNAs), to yield phase-specific gene expression or regulation patterns has been effective. Previous studies have revealed that RNA regulation mediates diverse biological processes, such as vascular remodeling (Fujiwara et al., 2021), innate immunity (Colgan et al., 2020), brain activity (Butt et al., 2021), and inflammation (Colgan et al., 2016), in plateau animals. Hence, insight into the underlying RNA regulatory mechanism in ATII cells of Tibetan pigs is of great significance for understanding hypoxic adaptation mechanisms. Here, we assumed that the expressions and functions of hypoxia-related genes are partially or entirely regulated by miRNAs or circRNAs. To validate this hypothesis, we constructed a competing endogenous RNA (ceRNA) network for ATII cell under normoxic (21% O_2_) and hypoxic (2% O_2_) conditions, which will allow identification of the changes in RNA regulation that occur in response to hypoxia in Tibetan pigs and Landrace pigs.

## Methods

### Sample collection

Lung tissue samples collected from healthy newborn male piglets (Tibetan pigs and Landrace pigs) at 7 days of age were soaked in PBS. Primary ATII cells were isolated as described previously (Wang et al., 2016) with minor modifications. Then, ATII cells were cultured in complete medium at 37°C in an environment containing either 21% O_2_, 5% CO_2_, and 79% N_2_ (normoxic conditions) or 2% O_2_, 5% CO_2_, and 98% N_2_ (hypoxic conditions).

We harvested ATII cells (n = 6 for each group) at 48 h under the 21% O_2_ (Tibetan–normoxic (TN) and Landrace–normoxic (LN)) or 2% O_2_ (Tibetan–low hypoxic (TL) and Landrace–hypoxic (LL)) conditions. Three of each group were flash-frozen in liquid nitrogen for RNA extraction, and the rest were used for analyze cell apoptosis by A BD FACSCanto II flow cytometer (BD Biosciences, San Jose, CA, USA).

### RNA isolation, library preparation, and sequencing

Total RNA was extracted with a TRIzol reagent kit (Invitrogen, Carlsbad, CA, USA), and the RNA integrity number (RIN) was confirmed to be >7.5. mRNA and noncoding RNA (ncRNA) were retained by removing ribosomal RNA (rRNA), and short fragments were reverse transcribed into complementary DNA (cDNA) with random primers. Second-strand cDNA was synthesized and purified with a QiaQuick PCR Extraction Kit (Qiagen, Venlo, Netherlands). Twelve RNA-seq libraries were generated and sequenced on the Illumina HiSeq™ 4,000 sequencing platform (Illumina Inc., San Diego, CA, USA) by Gene *Denovo* Biotechnology Co. (Guangzhou, China).

### Read mapping and transcript assembly

Clean reads were obtained by filtering adapter reads and poor-quality reads from the raw data using fastp (Chen et al., 2018). HISAT 2 was used to map the clean paired-end reads to the *Sus scrofa* RefSeq genome (*Sus scrofa* 11.1). StringTie (Pertea et al., 2015) was used to reconstruct transcripts and calculate expression abundances and variations as fragments per kilobase of transcript per million mapped reads (FPKM) values.

### Identification of known and novel miRNAs and circRNAs

Clean reads were obtained and mapped to the miRBase 21.0 database (http://www.mirbase.org/) to identify known porcine miRNAs. Other known miRNAs in other species were identified and mapped to the remaining miRNA sequences. Novel miRNA candidates were predicted by aligning the reference genome with unannotated tags according to hairpin structures and genomic positions using miRDeep2.

Clean reads were obtained and mapped to the porcine reference genome by discarding low-quality reads, and the retained reads were analyzed with find_circ to identify circRNAs. Clean reads that could not be annotated were defined as novel circRNAs.

### Quantification of RNA abundance and evaluation of differential expression

The expression of the identified circRNAs, miRNAs, and mRNAs was quantified by calculating the reads per million mapped reads (RPM), transcripts per million (TPM), and FPKM values, respectively. Differentially expressed miRNAs (DEmiRNAs) and differentially expressed circRNAs (DEcircRNAs) were identified as those with a fold change (FC) ≥2 and *p* value < 0.05 by edgeR, and differentially expressed mRNAs (DEmRNAs) were identified as those with a false discovery rate (FDR) of less than 0.05 and an absolute FC ≥ 2 by DESeq.

### Functional annotation and enrichment analysis of RNAs

Source genes of DEcircRNAs, candidate target genes of DEmiRNAs, and DEmRNAs were subjected to Gene Ontology (GO) term enrichment analysis (http://www.geneontology.org/) and Kyoto Encyclopedia of Genes and Genomes (KEGG) pathway enrichment analysis (http://www.genome.jp/kegg/). FDR ≤0.05 was established as the threshold for determining significance.

### Construction of the circRNA–miRNA–mRNA network

RNA pairs with Spearman rank correlation coefficients (SCCs) < −0.7 were selected as negatively coexpressed mRNA–miRNA or circRNA–miRNA pairs, and RNA pairs with Pearson correlation coefficients (PCCs) > 0.9 were selected as coexpressed circRNA–mRNA pairs. *p* values < 0.05 were computed to assess shared miRNA sponges between circRNAs and mRNAs as final ceRNA pairs. The circRNA–miRNA–mRNA ceRNA regulatory network was constructed and visualized using Cytoscape. We considered significant nodes at the cores of the regulated networks to be highly associated with hypoxic adaptation.

### Quantitative real-time–PCR validation

To verify the RNA-seq results, we randomly selected mRNAs, miRNAs, and circRNAs to verify the data reliability. The primers used for qRT–PCR were synthesized by Qingke Biological Company (Xi’an, China) and are shown (Supplementary Table S1 in Supplementary Material S2). cDNA was synthesized from RNA samples identified by transcriptome sequencing and used as the template for gene expression analysis. qPCR was performed in a LightCycler 96 Real-Time System (Roche, Switzerland) using SYBR^®^ Premix Ex Taq™ II (TaKaRa, China). Statistical analyses were performed with analysis of variance (ANOVA) using SPSS 20.0 (SPSS, IL, USA). *p* < 0.05 was considered to indicate a significant difference. Graphs were generated using GraphPad Prism 8.0 (GraphPad Software, CA, USA).

### Dual-luciferase reporter assay

The putative miR-141 binding sites on the target gene *HOOK3* were predicted using Target Scan (http://www.Targetscan.org), miRBase (http://www.mirbase.org), and miRDB (http://www.mirdb.org). The wild-type (WT) and mutant-type (MUT) *HOOK3* containing the putative binding site of miR-141 were synthesized by GENEWIZ Biotechnology Co., Ltd. and purchased from GENEWIZ (Suzhou, China) andcloned into the pmirGLO Dual-Luciferase vector XhoI/SalI sites (Promega, United States). The mimic negative control (NC) were bought by Jima (Shanghai, China). HOOK3-MT or HOOK3-MUT and miR-141 mimic or mimic NC were co-transfected into 293 T cells using Lipofectamine 2000 transfection vehicle (Invitrogen, Carlsbad, CA, United States) for the luciferase reporter assay. After 48 h, luciferase activity were examined by the Dual-Luciferase Reporter Assay System (Promega). The ratios of firefly luciferase to renilla luciferase were identified as relative luciferase enzyme activity.

## Results

### Correlation analysis of *COL5A1* and cell apoptosis

**FIGURE 1:**
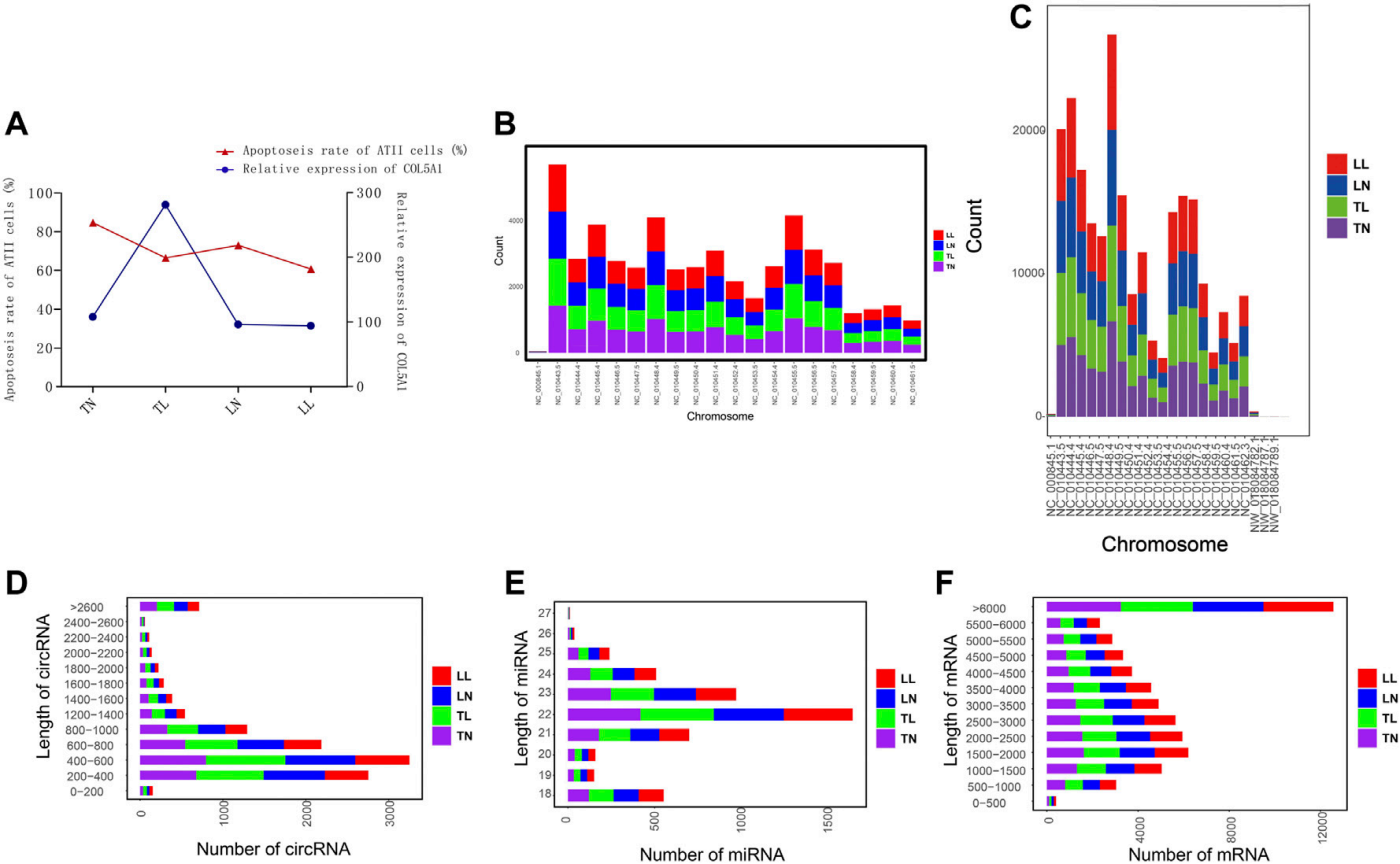
General features of circRNAs, miRNAs, and mRNAs. **(A)** Correlation analysis of COL5A1 and cell apoptosis. **(B)** Chromosome distribution map of circRNAs. **(C)** Chromosome distribution map of mRNAs. **(D–F)** Length distributions for the identified circRNAs, miRNAs, and mRNAs.

Cell apoptosis was investigated by flow cytometric assays, and our results showed that cell apoptosis was higher in the hypoxic groups (TL, LL) than that in the normoxic groups (TN, LN), and the rate of normal viable cell rate were 84.63, 66.47, 72.95, and 60.68% in TN, TL, LN, LL groups, respectively (Table 1). Notably, the expression of *COL5A1* in TL were significant higher than that of TN groups, and not significant different between LN and LL groups, as the source genes of numerous circRNAs, including novel_circ_001282, novel_circ_001569, and novel_circ_003568 (Figure 1A).

**TABLE 1:** Apoptosis rate of ATⅡ cells under hypoxia.

Groups	Late apoptosis rate (%)	Early apoptosis rate (%)	Total rate of apoptosis (%)	Normal viable cell rate (%)
LN	19.78	4.45	24.23	72.95
LL	29.98	6.89	36.87	60.68
TN	7.29	6.58	13.87	84.63
TL	15.40	16.50	31.90	66.47

### Identification of DEcircRNAs, DEmiRNAs, and DEmRNAs

**FIGURE 2:**
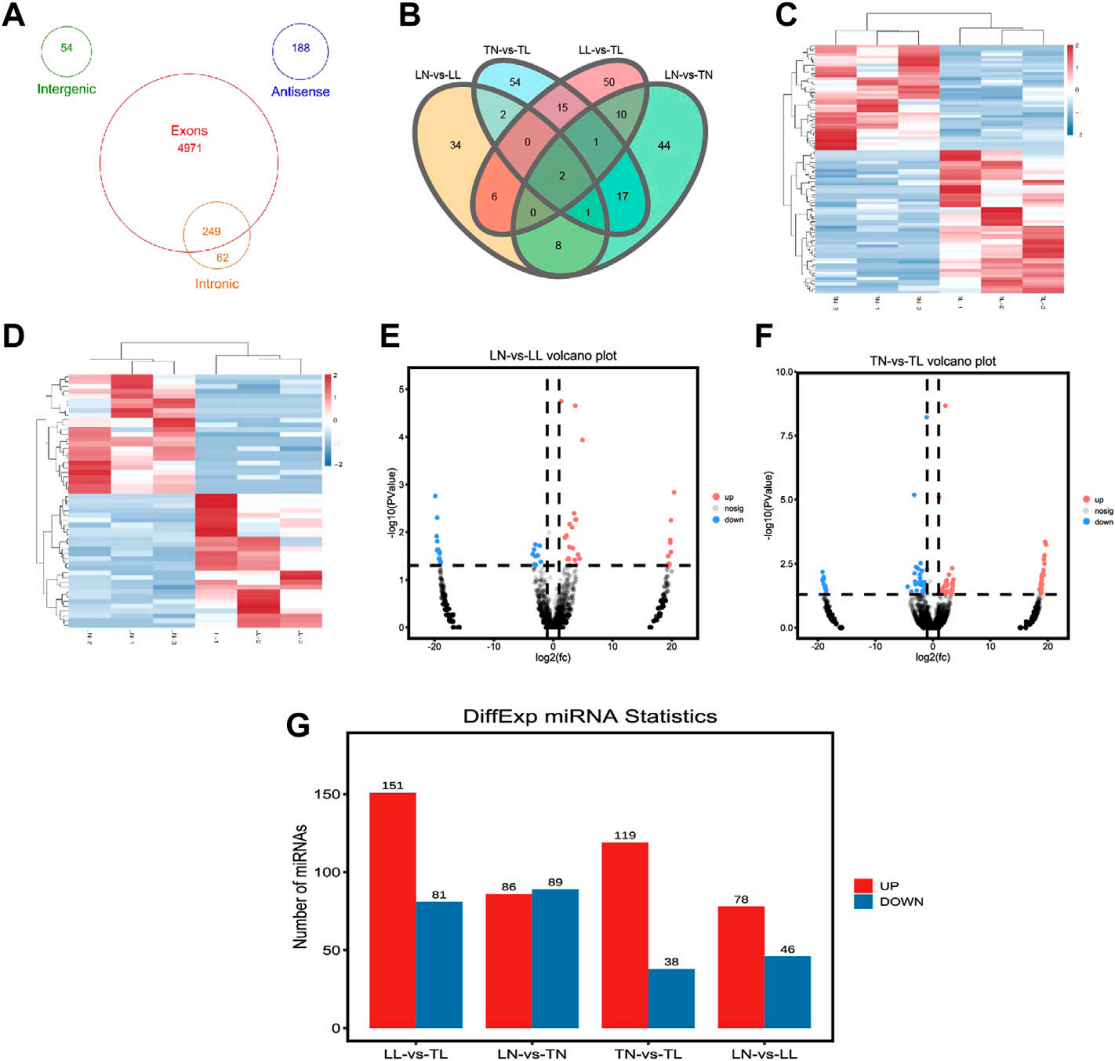
Summary of differential expression analysis of the circRNAs and miRNAs. **(A)** Venn diagram indicating that circRNAs numbers originated from different genomic sites. **(B)** Venn diagram of circRNAs interactions based on the overlapping circRNAs among the four groups. **(C)** Heatmap showing the relative expression patterns of DEcircRNAs between TN and TL. **(D)** Heatmap showing the relative expression patterns of DEcircRNAs between LN and LL. **(E)** Volcano plot showing the relative expression patterns of DEcircRNAs between LN and LL. **(F)** Volcano plot showing the relative expression patterns of DEcircRNAs between TN and TL. **(G)** Histogram showing the number of DEmiRNAs identified among the four groups.

Whole-transcriptome profiling of ncRNAs (i.e., circRNAs), mRNAs, and miRNAs was performed to assess the regulation of hypoxia-related genes in ATII cells (Supplementary Figure S1 in Supplementary Material S1, Supplementary Tables S2, S3 in Supplementary Material S2). Averages of 74.97 and 11.89 million clean reads obtained for circRNAs (mRNAs) and miRNAs, respectively, after removal of redundant and low-quality reads. mRNAs and circRNAs were identified on all chromosomes; most mRNAs were located on chromosomes NC_010448.4, NC_010444.4, and NC_010443.5, and most circRNAs were located on chromosomes NC_010443.5, NC_010455.5, and NC_010448.4 (Figures 1B,C). In total, 5,524 circRNAs were identified in this study, and these were named and numbered novel_circ_000001 to novel_circ_005524. Furthermore, the vast majority of these circRNAs were shown to have originated from exonic circRNAs, and intronic, intergenic, antisense overlapping and sense overlapping circRNAs were the mainly kind of circRNAs identified (Figure 2A). Most of the circRNAs, miRNAs, and mRNAs had lengths of 200–1,000 bp, 21–24 bp, and more than 1,000 bp, respectively; 400–600 bp, 22–23 bp, and more than 6,000 bp were the most abundant respective lengths and were consistent with the RNA features (Figures 1D–F).

**FIGURE 3:**
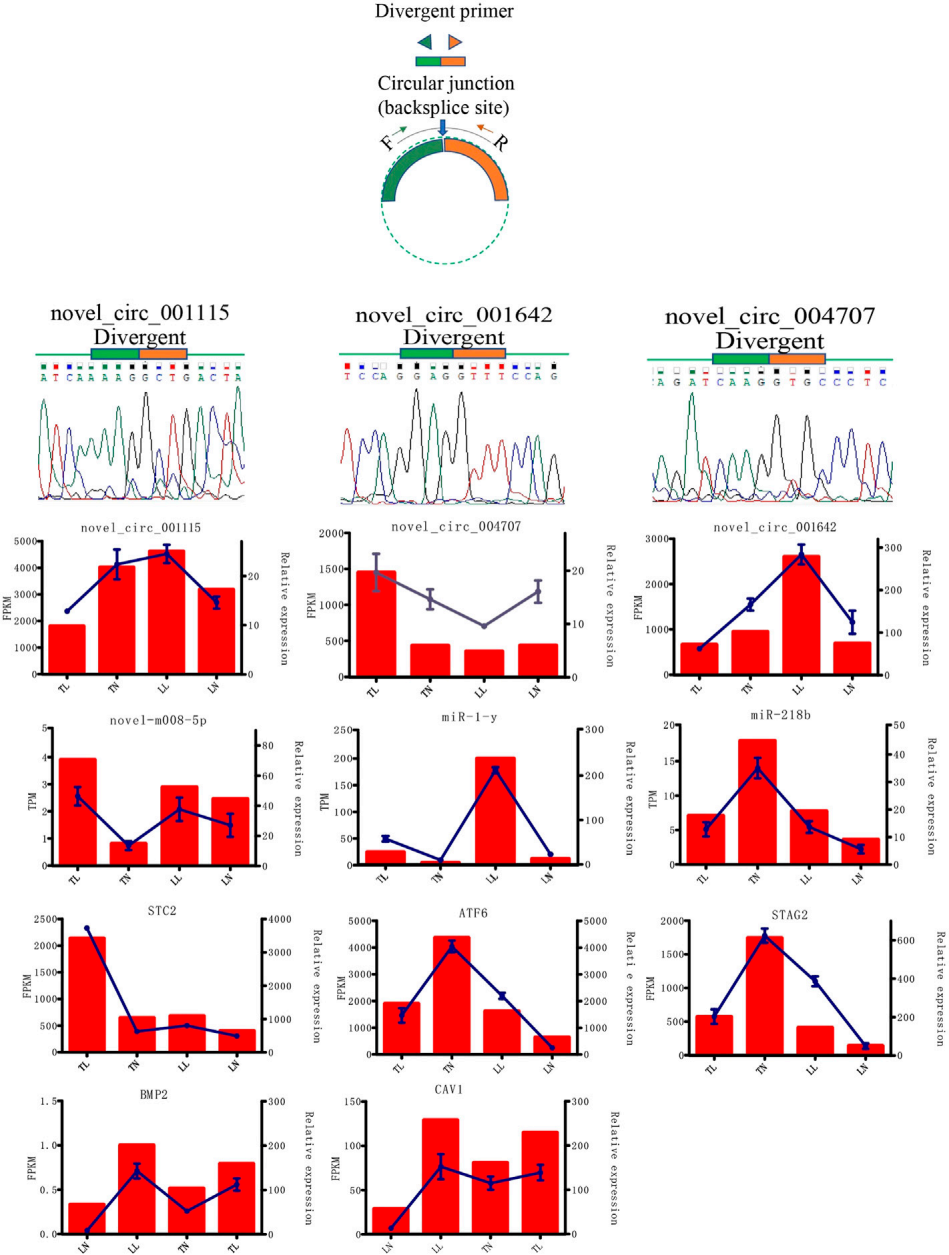
Expression patterns of randomly selected 3 DEcircRNAs, 3 DEmiRNAs and 3 DEmRNAs.

In total, 5,524 circRNAs, 1,332 miRNAs, and 20,720 mRNAs were obtained. A total of 92 (53 upregulated and 39 downregulated), 53 (28 upregulated and 25 downregulated), 83 (47 upregulated and 36 downregulated), and 84 (49 upregulated and 35 downregulated) DEcircRNAs were identified in ATII cells between the TN and TL, LN and LL, TN and LN, and TL and LL groups, respectively (Table 1; Figures 2B–F). Analysis identified 340 existing miRNAs, 675 known miRNAs, and 318 novel miRNAs (Supplementary Material S3). In addition, 5 DEcircRNAs, 37 DEmiRNAs, and 1,470 DEmRNAs were identified between cells cultured under different oxygen conditions (21% O_2_ and 2% O_2_) (Supplementary Material S4). The 3 DEcircRNAs, 3 DEmiRNAs, and 3 DEmRNAs randomly selected and detected by qPCR analysis and Sanger sequencing to validate the accuracy of the sequencing data. Result suggest that the sequencing data and expression of circRNAs, miRNAs, and mRNAs identified in this study are reliable (Figure 3, Supplementary Figure S2 in Supplementary Material S1).

### Identification of miRNA–mRNA and circRNA–miRNA pairs

A total of 157 and 124 hypoxia-dependent DEmiRNAs were identified in the Tibetan pig (TN and TL) groups and Landrace pig (LN and LL) groups (Figure 2G), respectively. Furthermore, we predicted the target circRNAs and mRNAs of these DEmiRNAs and identified 109 (25) circRNA–miRNA and 25,434 (18,506) mRNA-miRNA pairs between the TN (LN) and TL (LL) groups (Supplementary Material S5).

### GO and KEGG enrichment analyses of DEcircRNA source genes

**FIGURE 4:**
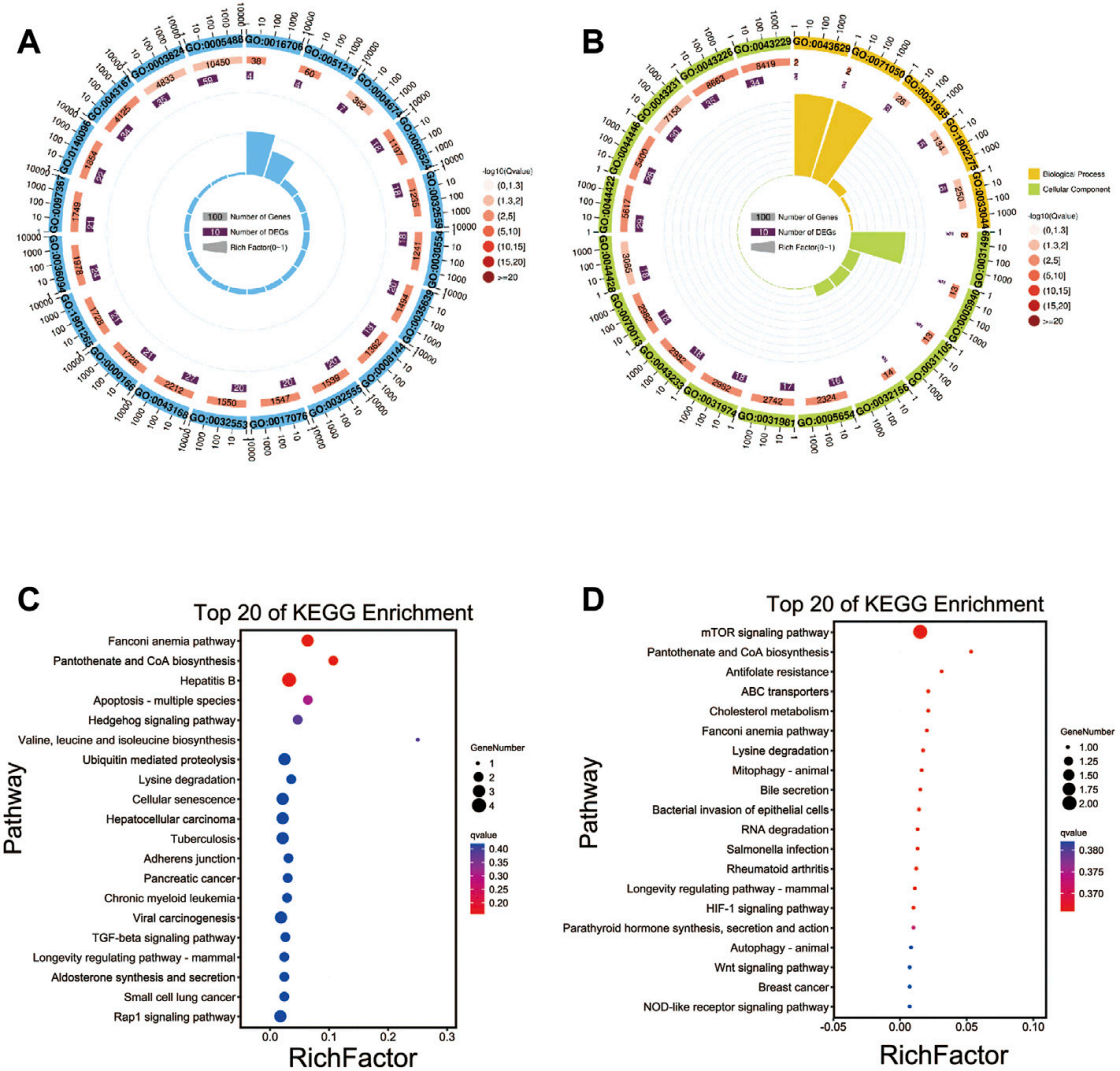
Functional annotation analysis of the source genes of DEcircRNAs. **(A)** GO annotation of the source genes of DEcircRNAs between TN and TL groups. **(B)** GO annotation of the source genes of DEcircRNAs between LN and LL groups. **(C)** KEGG enrichment of the source genes of DEcircRNAs between TN and TL groups. **(D)** KEGG enrichment of the source genes of DEcircRNAs between LN and LL groups.

We found that the majority of DEcircRNAs among the four groups were derived from source genes and that some were derived from intergenic regions. We also performed GO functional analysis and KEGG pathway enrichment analysis of the circRNA source genes to evaluate their biological roles. In total, 41, 38, 45, and 47 GO terms for the TN vs. TL, LN vs. LL, LN vs. TN, and LL vs. TL comparisons, respectively, were enriched in biological process, cellular component, and molecular function categories (Supplementary Figure S3A in Supplementary Material S1). Moreover, anion binding (GO:0043168), small molecule binding (GO:0036094), and ATP binding (GO:0005524) were the most significant terms between the TN and TL groups (Figure 4A). ncRNA polyadenylation (GO:0043629), snoRNA polyadenylation (GO:0071050), and TRAMP complex (GO:0031499) were the most significant terms between the LN and LL groups (Figure 4B).

KEGG enrichment analysis revealed that several pivotal pathways, including protein processing in the endoplasmic reticulum (ER), focal adhesion, and adherens junction, were associated with the regulatory mechanisms of the circRNAs (Supplementary Figure S3B in Supplementary Material S1). The source genes of the DEcircRNAs in the TN group compared to the TL group were significantly enriched in the Fanconi anemia pathway, pantothenate and CoA biosynthesis, and hepatitis B pathways (Figure 4C). The source genes of the DEcircRNAs in the LN group compared to the LL group were significantly enriched in the mTOR signaling pathway and pantothenate and CoA biosynthesis pathways (Figure 4D).

### Enrichment analysis of target genes of the DEmiRNAs in the network

**FIGURE 5:**
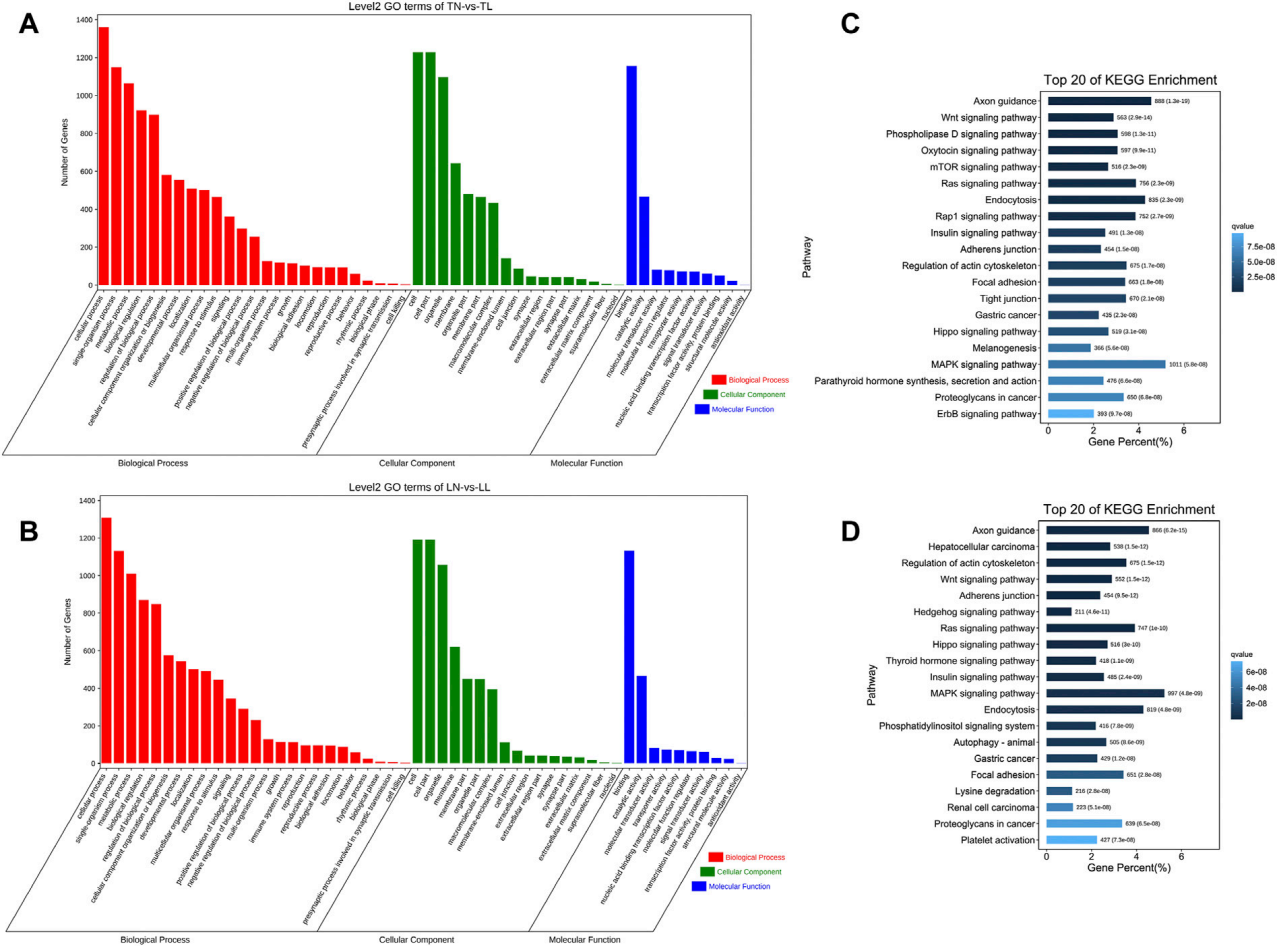
Functional annotation analysis of the target genes of DEmiRNAs. **(A)** GO annotation of the target genes of DEmiRNAs between TN and TL groups. **(B)** GO annotation of the target genes of DEmiRNAs between LN and LL groups. **(C)** KEGG enrichment of the target genes of DEmiRNAs between TN and TL groups. **(D)** KEGG enrichment of the target genes of DEmiRNAs between LN and LL groups.

We performed GO enrichment analysis to investigate the biological roles of target genes of DEmiRNAs and found that these genes were significantly enriched in the terms binding, intracellular, and intracellular part under hypoxic conditions. These terms were identified by comparison of the normoxia (21% O_2_) and hypoxia (2% O_2_) groups and represent the common effect of oxygen concentration changes on target genes of the DEmiRNAs (Supplementary Material S6). Most target genes of the DEmiRNAs between the TN and TL groups were significantly enriched in the cell term in the cellular component category, the binding term in the molecular function category, and the cellular component organization term in the biological process category (Figure 5A). Numerous target genes of the DEmiRNAs between the LN and LL groups were significantly enriched in the cellular component organization, nucleoside binding, and membrane-bounded organelle terms in the biological process, molecular function, and cellular component categories, respectively (Figure 5B).

As demonstrated by the KEGG pathway enrichment analysis, target genes of the DEmiRNAs between Tibetan pigs and Landrace pigs at different oxygen concentrations (2% O_2_ and 21% O_2_) were significantly enriched in the following pathways: axon guidance, focal adhesion, and MAPK signaling pathway (Supplementary Material S6). DEmiRNAs in Tibetan pigs at different oxygen concentrations were significantly enriched in the following pathways: axon guidance, wnt signaling pathway, and phospholipase D signaling pathway (Figure 5C). DEmiRNAs in Landrace pigs at different oxygen concentrations were significantly enriched in the following pathways: axon guidance, hepatocellular carcinoma, and regulation of actin cytoskeleton (Figure 5D).

### Establishment of the circRNA–miRNA–mRNA network

**FIGURE 6:**
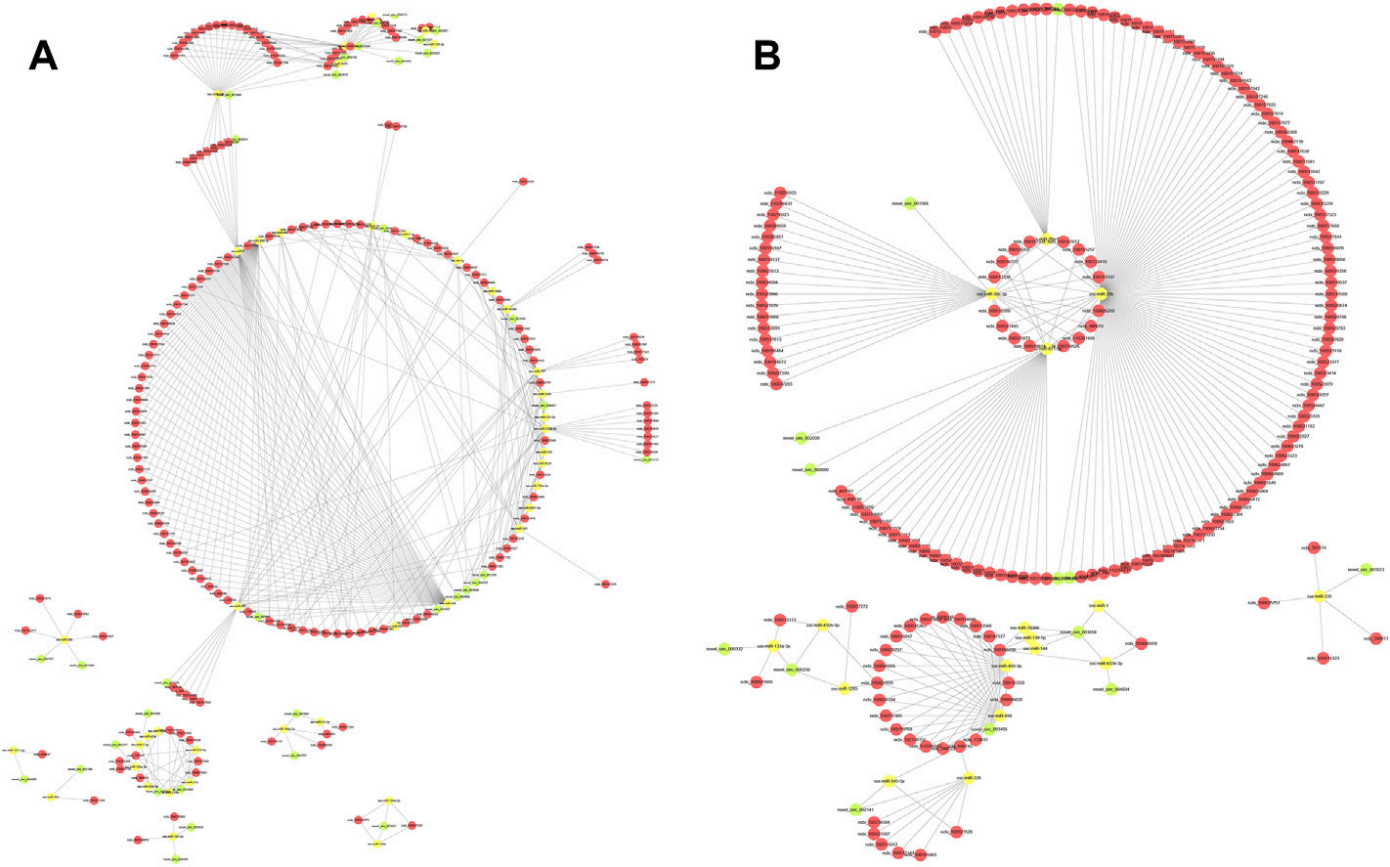
The circRNA-miRNA-mRNA interaction network. **(A)** The circRNA-miRNA-mRNA interaction network between TN and TL. **(B)** The circRNA-miRNA-mRNA interaction network between LN and LL. Green circles represent circRNAs, yellow circles represent miRNAs, and red circles represent mRNAs.

The intersection of circRNA–miRNA pairs and mRNA–miRNA pairs was filtered, and pairs based on the DEmiRNAs were identified in the TN vs. TL and LN vs. LL comparisons (Supplementary Material S7). To further reveal the potential regulatory networks and their biological processes in ATII cells at different oxygen concentrations, we combined the miRNA–mRNA and circRNA–miRNA pairs to construct two preliminary circRNA–miRNA–mRNA networks in Tibetan pigs and Landrace pigs. The network in Tibetan pigs presented an initial view of the associations among the 53 circRNA nodes, 44 miRNA nodes, 1,375 mRNA nodes, and 428 edges and included ssc-let-7g, ssc-miR-1, ssc-miR-10382, novel_circ_000083, novel_circ_000136, novel_circ_000480, *ENPP1*, *EPAS1*, *ITGB8*, etc. Ssc-miR-1, ssc-miR-490–3p, ssc-miR-490, and novel_circ_004392 were considered the most notable RNAs between the TN and TL groups (Figure 6A).

The network in Landrace pigs (LN vs. LL) was composed of 14 circRNA nodes (novel_circ_000256, novel_circ_000332, novel_circ_000880, etc.), 19 miRNA nodes (ssc-miR-1, ssc-miR-326, ssc-miR-335, etc.), 827 mRNA nodes (*EPAS1*, *NREP*, *P4HA1*, etc.) and 244 edges. Interestingly, ssc-miR-30c-3p, ssc-miR-20b, ssc-miR-671–5p, ssc-miR-29a-5p, and novel_circ_003405 were identified as the most important and central RNAs between the LN and LL groups (Figure 6B).

### Core regulatory networks of the DEcircRNAs, DEmiRNAs, and DEmRNAs

**FIGURE 7:**
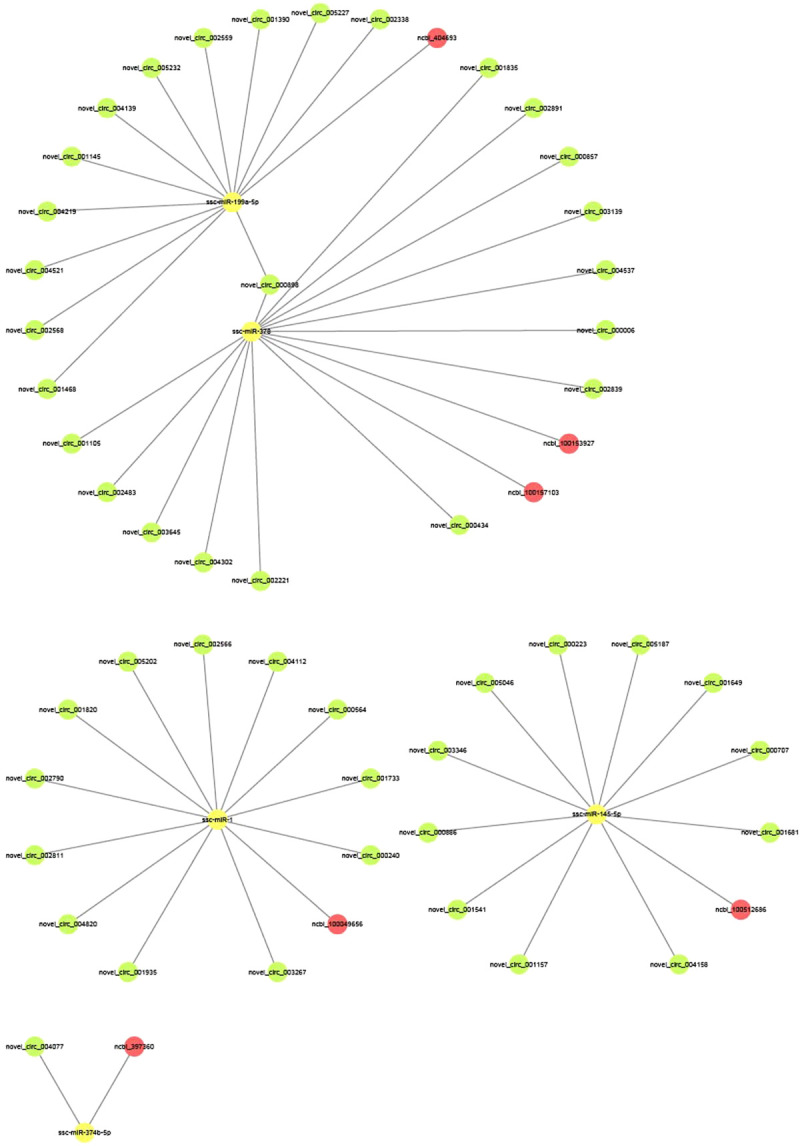
The circRNA-miRNA-mRNA interaction network. Green circles represent circRNAs, yellow circles represent miRNAs, and red circles represent mRNAs.

We combined the circRNA–miRNA and miRNA–mRNA pairs among the four groups and constructed a preliminary circRNA–miRNA–mRNA network. The top 50 relationship pairs are shown in the network diagrams. The network was composed of 49 circRNA nodes (novel_circ_001145, novel_circ_001390, novel_circ_001681, etc.), 5 miRNA nodes (ssc-miR-145–5p, ssc-miR-1, ssc-miR-378, etc.), 6 mRNA nodes (*MAPK1*, *IRS1*, *CAV1*, etc.) and 170 edges. Several miRNAs, such as ssc-miR-199a-5p, ssc-miR-378, and ssc-miR-1, were identified as the most important nodes, and related circRNAs (novel_circ_000857, novel_circ_001835, and novel_circ_001145) and mRNAs (ncbi_404,693, ncbi_100153927, NewGene.10854.1, and ncbi_100157103) were identified by miRanda (Figure 7).

### Validation of targeting relationships between miR-141 and *HOOK3*

Based on the inverse expression trends between miRNA and mRNAs in type II alveolar epithelial cells under different altitude. We randomly selected miR-141 to verify it targeting relationships with *HOOK3*. The dual luciferase reporter assay indicated that the luciferase activity was significantly decreased following co-transfection with the miR-141 mimic and HOOK3-WT (*p* < 0.01), while no effect on the mutant types of HOOK3-MUT (Supplementary Figure S4 in Supplementary Material S1). These results initially confirmed the direct interactions between miR-141 and *HOOK3*.

## Discussion

Tibetan pigs have historically lived in hypoxic environments and have undergone strong selection, and their molecular patterns may represent regulatory mechanisms of adaptation to high-altitude hypoxia (Li et al., 2013; Yang et al., 2021b). Accumulating research indicates that circRNAs, miRNAs, and mRNAs are involved in various types of physiological responses to hypoxia, including glycolysis (Fuller et al., 2020), aging (Haque et al., 2020), and cancer (Pan et al., 2021). The critical roles of mRNAs (Sahu et al., 2019), miRNAs (Hoefel et al., 2019) and circRNAs (Xu et al., 2020) are well known for lung development and disease prevention. Since 2013, an increasing number of researchers have focused on explaining the novel functions of circRNAs based on their tissue-specific expression and high stability as potential biomarkers (Shi et al., 2020; Khan et al., 2021). Occasionally, ATII cells can proliferate and differentiate into ATI cells after the death of them, which are responsible for gas exchange and serve as a barrier that participates in pathogen defense (Aspal and Zemans, 2020). Understanding the circRNA–miRNA–mRNA axis in ATII cells of Tibetan pigs and Landrace pigs could provide important insights into the protective mechanisms of the hypoxia response. The emerging hypoxia regulation function of circRNAs is particularly interesting, as they might thus be candidates for new therapeutic targets and biomarkers for disease. In this study, we identified and characterized the expression patterns of 5,524 circRNAs, 1,332 miRNAs, and 20,720 mRNAs in ATII cells of Tibetan pigs and Landrace pigs via high-throughput sequencing, further explored the circRNA–miRNA–mRNA regulatory axis and provided new insights into the regulatory roles of RNAs in hypoxia.

### Hypoxia may activated DNA repair and damage pathways

To investigate the roles of circRNAs in the response of ATII cells to hypoxia, functional analysis of their source genes was performed. DNA repair and damage pathways may be activated during replication stress in cells under hypoxia. GO functional enrichment analysis revealed that the circRNA source genes participated mainly in biological processes, including cell proliferation, cellular processes, and cell killing. Most DEcircRNAs between the LN and LL groups (such as novel_circ_003062, novel_circ_004904, and novel_circ_003307) were generated from DNA damage-related genes, such as *ZNF*451, *TLK*1, and *TET*1, indicating the presence of DNA damage in ATII cells of Landrace pigs under hypoxic conditions, leading to the activation of numerous distinct repair mechanisms and signaling pathways. The small ubiquitin-related modifier (SUMO) ligase ZATT (*ZNF*451) can control cellular responses to topoisomerase 2-mediated damage as a multifunctional DNA repair factor (Schellenberg et al., 2017). Mammalian *TLK*1 was identified in 1999 and is involved in chromatin formation and newly replicated DNA processing as a serine/threonine kinase; in turn, it can be phosphorylated and deactivated by Chk1 in response to DNA damage under hypoxia (Silljé et al., 1999; Krause et al., 2003). Ten-eleven translocation-1 (TET1) mediates the influence of hypoxia on modifying adipocytokine DNA methylation, and its DNA demethylase activity is induced by *HIF*-*1α* (Ali et al., 2020). In addition, oncogenic *TNKS*2 can promote the migration and invasion of cervical cancer cells by directly upregulating miR-20a (Kang et al., 2012). *KLF*12 is a member of the Krüppel-like factor family, and its overexpression directly affects proangiogenic processes via transcriptional regulators participating in a multitude of cancer-relevant processes (Mao et al., 2020). Several DEcircRNAs between Landrace pigs in the LN and LL groups (such as novel_circ_001713, novel_circ_000707, and novel_circ_005275) were derived from cancer- and disease-related genes, such as *TNKS*2, *KLF*12, and *BTAF*1, which activate numerous signaling pathways involved in DNA damage, apoptosis promotion, and mitochondrial dysfunction caused by an insufficient oxygen supply (Adami et al., 2018; Vinchure et al., 2021).

### ATII cell apoptosis may effect a modulator of cell repair in tibetan pigs under hypoxia

In the TN and TL groups, DEcircRNAs (such as novel_circ_000961, novel_circ_004860, and novel_circ_004404) were generated from inhibitors of apoptosis proteins (e.g., baculoviral IAP repeat containing 6 (*BIRC*6)), which may lead to a key autonomous effect as a modulator of cell repair in Tibetan pigs under hypoxia (Guo et al., 2019). Moreover, we suspect that *BIRC*6 could inhibit apoptosis by facilitating ubiquitination-mediated degradation of apoptotic proteins (Hao et al., 2020). In contrast, *ATK*17*A* and *CASP*2 exhibit apoptosis-inducing activity and can mediate cellular apoptosis (Okumu et al., 2017). DEcircRNAs between the TN and TL groups (such as novel_circ_005192, novel_circ_003369, and novel_circ_000923) were derived from *PLOD*2, which may modulate the migration and invasion of cells via PI3K/Akt signaling (Wan et al., 2020). DEcircRNAs between the TN and TL groups (such as novel_circ_001296, novel_circ_001418, novel_circ_000169) were derived from *PARD*3, which may be associated with asymmetrical cell division and direct polarized cell growth. *PARD*3 contains multiple postsynaptic densities and is localized to tight junctions; in addition, it is correlated with invasion in lung squamous cell carcinoma via impaired STAT3 signaling (Ohno, 2001; Ozdamar et al., 2005; Bonastre et al., 2015). As expected, we found that DEcircRNAs between the TN and TL groups (such as novel_circ_001282, novel_circ_001569, and novel_circ_003568) were derived from *COL5A1*, which encodes an alpha chain for one of the low-abundance fibrillar collagens and may regulated in a hypoxia- and SMC-NFATc3-dependent manner in Tibetan pigs during the response to hypoxia (Sheak et al., 2020). The significant higher expression of *COL5A1 i*n TL groups may inhibited apoptosis of ATII cells in Tibetan pigs under lower oxygen concentration, may lead their better survive in the hypoxia environment than Landrace pigs (Liu et al., 2018).

### CircRNA-associated ceRNA networks of ATII cells under hypoxia

Ssc-miR-136–5p, ssc-miR-490–3p, ssc-miR-1, ssc-miR-490, ssc-miR-335, ssc-miR-2320–3p, and ssc-miR-10390 were subsequently identified as the most affected miRNAs and were strongly correlated with numerous mRNAs and circRNAs in the ceRNA network based on DEmiRNAs between the TN and TL groups. CircTLK1 positively regulates CBX4 expression, promoting the proliferation and metastasis of renal cell carcinoma, by sponging miR-136–5p (Li et al., 2020). The novel_circ_002672–ssc-miR-136-5p–SOCS4 axis between the TN and TL groups may have a vital role in a series of pathological changes, including thyroid carcinoma, lung squamous cell cancer, pentose and glucuronate interconversion, and ascorbate, aldarate, and retinol metabolism; it may also regulate the IKKβ/NF-κB/A20 pathway as a modulator of the inflammatory response (Deng et al., 2018; Xie et al., 2018; Gao R. Z. et al., 2019). Among these miRNAs, altered expression of ssc-miR-136–5p, ssc-miR-490–3p, and miR-10390 target genes may occur in response to cellular stressors to facilitate the survival of ATII cells in Tibetan pigs (Jeffery and Harries, 2019).

Based on the ceRNA network between the LN and LL groups, ssc-miR-30c-3p, ssc-miR-20b, ssc-miR-671–5p, and ssc-miR-29a-5 could serve as central miRNAs to regulate the expression of numerous target circRNAs and mRNAs. The novel_circ_005449-ssc–miR-20b–*ZDBF*2 and novel_circ_000880–ssc-miR-671-5p–*CDC*14*B* axes are partial axes based on ssc-miR-20b and ssc-miR-671–5p, which are important regulators of 18 mRNAs, 1 circRNA and 21 mRNAs. Two DEcircRNAs were found in our network between the LN and LL groups, and these circRNAs may cause hypoxia injury-induced autophagy through autophagosomal degradation in Landrace pigs (Gao L. et al., 2019; Lu et al., 2020).

Multiple lines of evidence have demonstrated that circRNAs indirectly enhance gene expression by acting as miRNA sponges (Zhong et al., 2018). The overexpressed circRNA competitively binds to and inhibits the activity of a miRNA to prevent the degradation of the target mRNA. We further analyzed the circRNA–miRNA–mRNA network based on ceRNAs and identified ssc-miR-199a-5p, ssc-miR-378, ssc-miR-1, and ssc-miR-145–5p. Ssc-miR-199a-5p and ssc-miR-378 may regulate many miRNAs and mRNAs, including novel_circ_000898-ssc-miR-199a-5p-*CAV*1 and novel_circ_000898-ssc-miR-378-*BMP*2, which could mediate ER stress-induced apoptosis and inflammation and attenuate hypoxia-induced injury in ATII cells under hypoxic conditions (Wen et al., 2020). These findings may explain why ATII cells from Tibetan pigs can survive better than those from Landrace pigs under hypoxic conditions.

## Conclusion

In conclusion, we present comprehensive documentation elucidating the changes in and regulation of circRNA–miRNA–mRNA axes in ATII cells of Tibetan pigs and Landrace pigs under hypoxia induction. We identified several hypoxia-related genes and pathways showing adaptive changes in ATII cells, including cell proliferation, cellular processes, and cell killing. Our results also provide new insights into the response of ATII cells in Tibetan pigs to hypoxia, and the numerous identified circRNA miRNAs and mRNAs will serve as references for further investigation of their functions.

## Data Availability

The datasets presented in this study can be found in online repositories. The names of the repository/repositories and accession number(s) can be found in the article/Supplementary Material.

## Abbreviations

DEcircRNAs, Differentially expressed circRNAs; DEmiRNAs, Differentially expressed miRNAs; DEmRNAs, Differentially expressed mRNAs; TN, ATII cells of Tibetan pigs were cultured under 21% O_2_; TL, ATII cells of Tibetan pigs were cultured under 2% O_2_; LN, ATII cells of Landrace pigs were cultured under 21% O_2_; LL, ATII cells of Landrace pigs were cultured under 2% O_2_.
