# Supplementary material for: Characterization of circRNA–miRNA–mRNA networks regulating oxygen utilization in type II alveolar epithelial cells of Tibetan pigs
Source: Front Mol Biosci. 2022 Sep 21;9:854250. doi: 10.3389/fmolb.2022.854250 (PMC9532862; doi:10.3389/fmolb.2022.854250)
Supplement: Supplementary file 4 [file DataSheet1.doc]

**
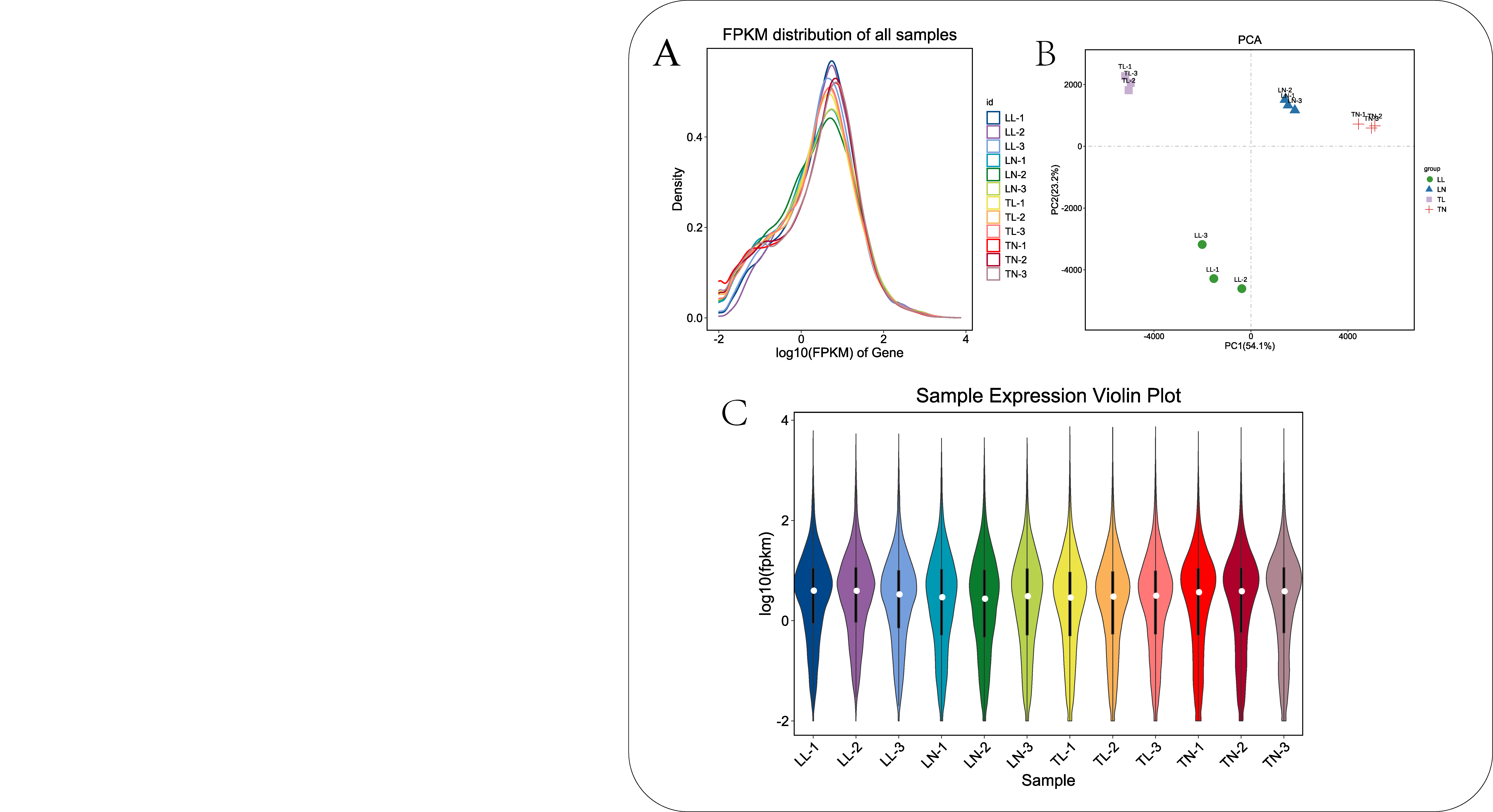
**

**Figure S1** A. Density plot of mRNA interactions based on the overlapping mRNAs among the four groups. B. A PCA score plot showed the distributions of mRNAs. C. Differential expression of mRNAs among four groups. P-values and log2FC values were used to screen for differentially expressed transcripts according to the following thresholds: P < 0.05 and |log2FC| > 1.


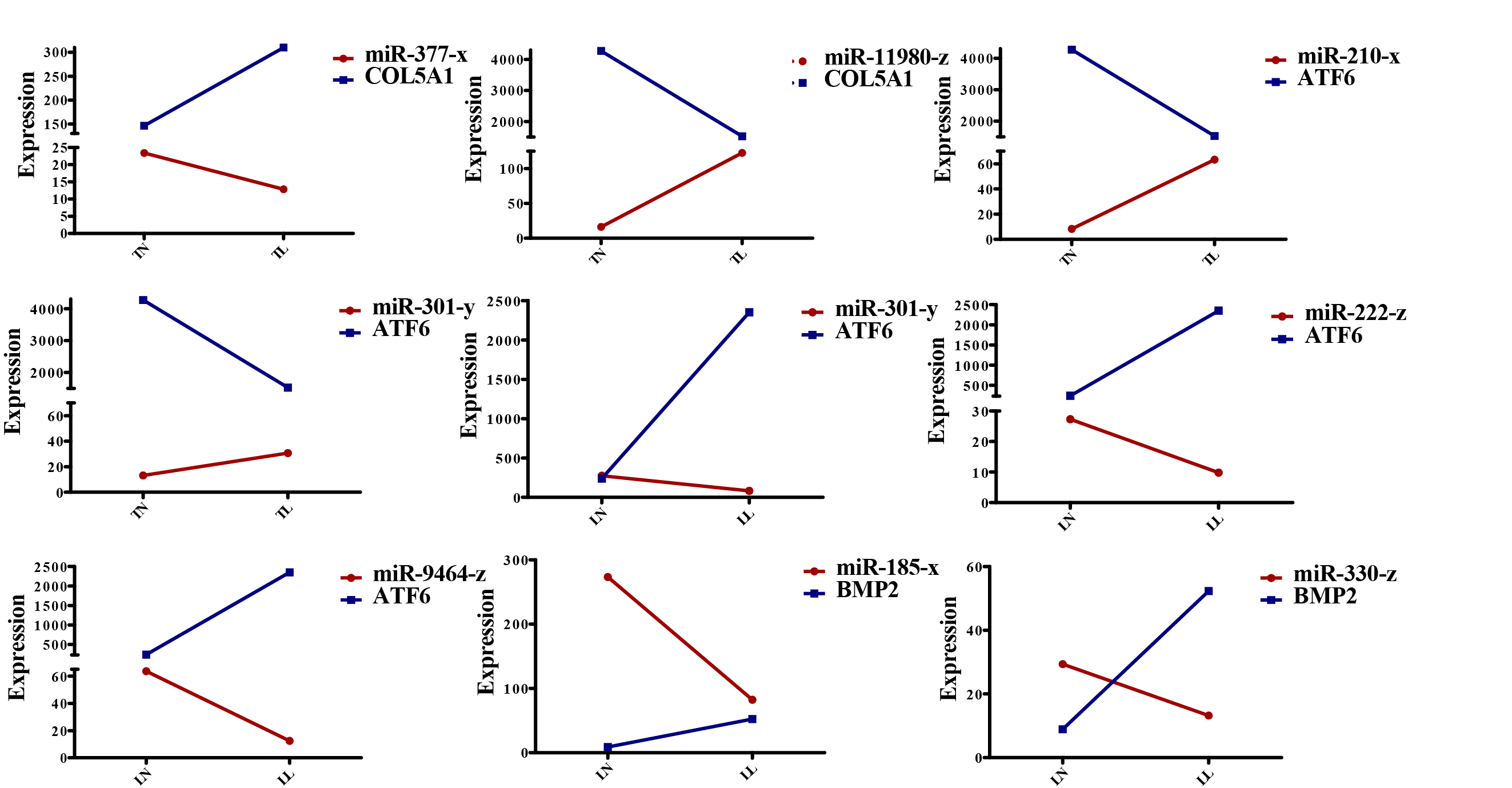


**Figure S2** Expression patterns of mRNAs and their target miRNAs.


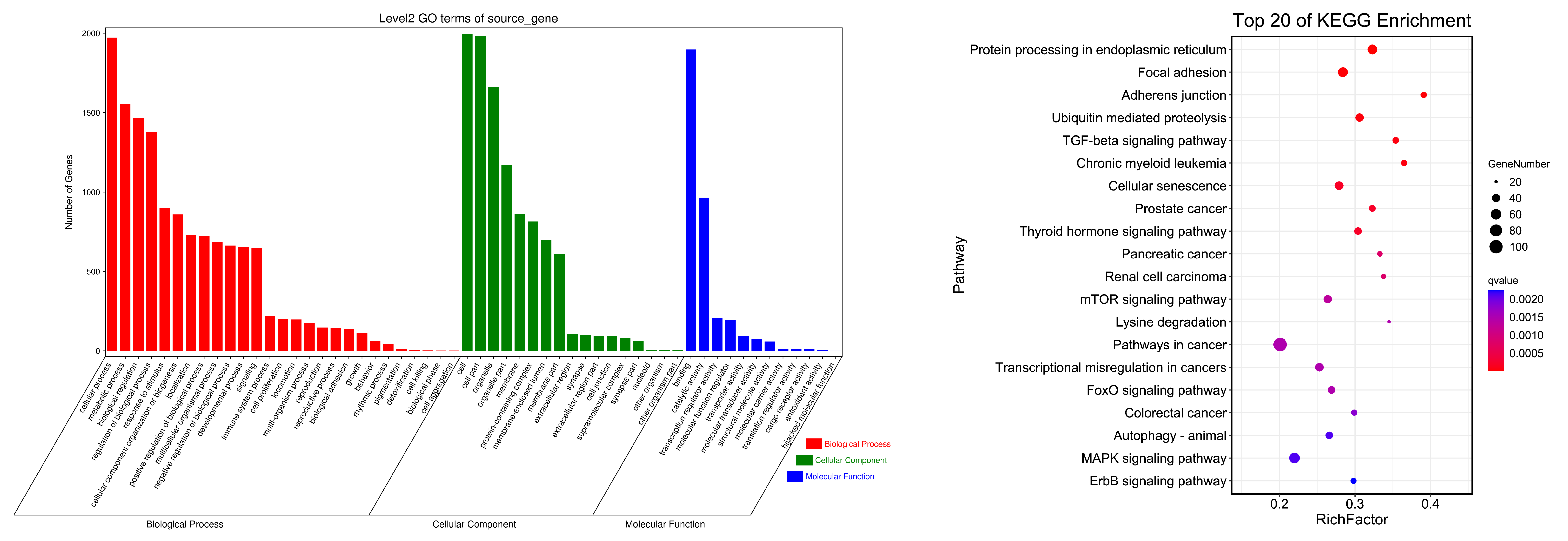


**Figure S3** Functional annotation analysis of the source genes of DEcircRNAs. (A) GO annotation of the source genes of DEcircRNAs among four groups. (B) KEGG enrichment of the source genes of DEcircRNAs among four groups.


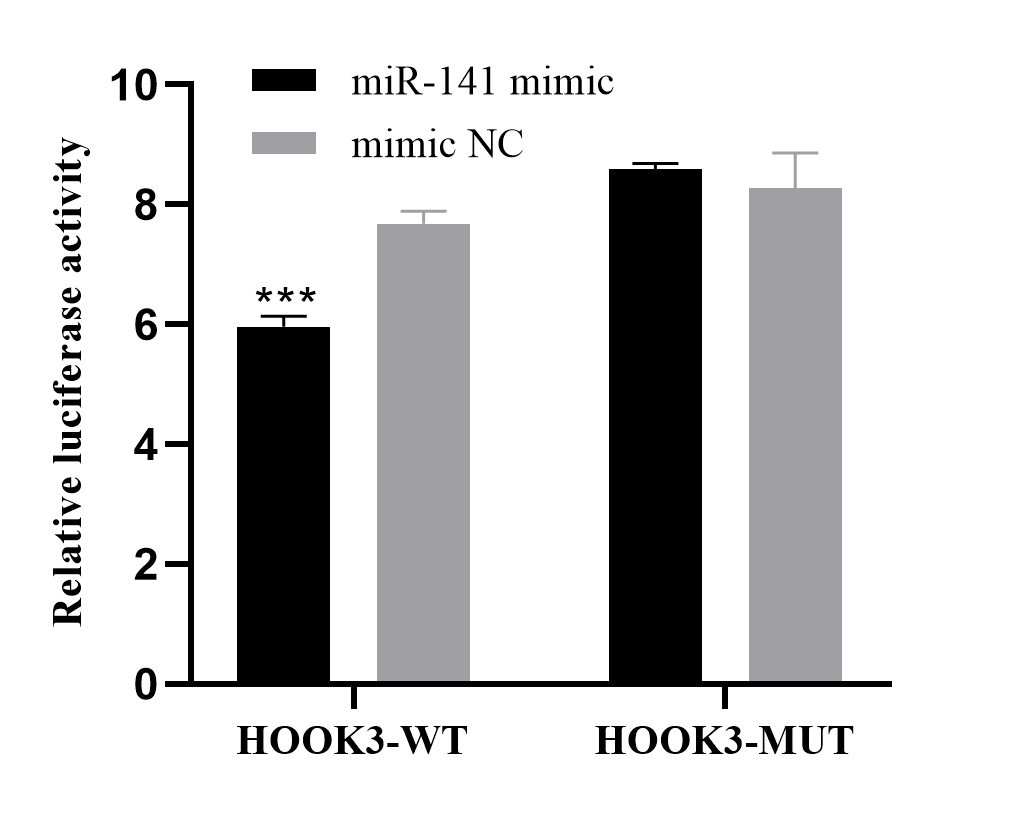


**Figure S4** Dual-luciferase assays were used to validate the binding sites between miR-141 and HOOK3.
