# Supplementary material for: Characterization of circRNA–miRNA–mRNA networks regulating oxygen utilization in type II alveolar epithelial cells of Tibetan pigs
Source: Front Mol Biosci. 2022 Sep 21;9:854250. doi: 10.3389/fmolb.2022.854250 (PMC9532862; doi:10.3389/fmolb.2022.854250)
Supplement: Supplementary file 12 [file DataSheet2.doc]

Table S1 Primers used to detect DEmRNAs, DEmiRNAs, and DEcircRNAs in ATII cells of pigs by qRT-PCR

| **Genes** | **Primer sequences (5'-3')** | **Annealing temperature/℃** |
| --- | --- | --- |
| novel_circ_001115 | F: GCCGTCACTCACCAACTCC | 60 |
|  | R: CCTTTTGATAAACAATGGAGCA |  |
| novel_circ_004707 | F: CAACGACTTGAAAACAAATGAC | 60 |
|  | R: GACTGGGAGGTTTGGGATT |  |
| novel_circ_001642 | F: GTCTCCAACATCATCACCTCTT | 60 |
|  | R: CTGGGGTCATGGCGAAT |  |
| novel_m008_5p | CAACCCGGTCAGCCTCC | 60 |
| miR-1-y | CCGGCGTGGAATGTAAAGAAGTATGT | 60 |
| ssc-miR-218b | CGCTTGTGCTTGATCTAACCATGTG | 60 |
| miR-377-x | GTTGCCCTTGGTGAATTCT | 60 |
| miR-11980-z | TAAGAACGGGCTTGGCTTA | 60 |
| miR-210-x | ATAATGCCCACCGCACAT | 60 |
| miR-301-y | GGGTGCAATGATATTGTCAA | 60 |
| miR-185-x | TGGAGAGAAAGGCAGTTCA | 60 |
| miR-330-z | TATACACGGCCTGCAGAGA | 60 |
| miR-222-z | AGCTACATCTGGCTACTGGG | 60 |
| miR-9464-z | GAGAAGATTTTGACAAGGGC | 60 |
| STC2 | F: CCAATCCGCCCGAAAGT | 60 |
|  | R: CACGCCACAGCCCACAT |  |
| ATF6 | F: CAGCCCAAGCCTTTGTTAC | 60 |
|  | R: CACCACCGTCTGACCTTTAGT |  |
| STAG2 | F: TGAAAAGAAGGGGACGACA |  |
|  | R: TGCTATCCACAAGGTATGCTG |  |
| β-actin | F: CAGTCGGTTGGATGGAGCAT  R: AGGCAGGGACTTCCTGTAAC | 60 |
| U6 | F: GGAACGATACAGAGAAGATTAGC | 60 |
|  | R: TGGAACGCTTCACGAATTTGCG |  |

Table S2 Overview of the reads and quality control of the 12 libraries of mRNA and circRNA sequencing from ATII cells

| Sample | RawData(bp) | BF_Q20 | BF_Q30 | BF_N | BF_GC | CleanData(bp) |
| --- | --- | --- | --- | --- | --- | --- |
| LL-1 | 9360523800 | 9158336486 | 8793276058 | 1148544 | 4245933596 | 9130040100 |
| LL-2 | 3844967700 | 3771913314 | 3636570451 | 1578555 | 1742923858 | 3676299300 |
| LL-3 | 6975536100 | 6818586538 | 6561189256 | 1685562 | 3221302571 | 6726007800 |
| LN-1 | 9269896500 | 9051126943 | 8687747000 | 423797 | 4389295993 | 8997964200 |
| LN-2 | 9584649000 | 9341198915 | 8946311377 | 426774 | 4569960643 | 9324353400 |
| LN-3 | 10133538900 | 9894387382 | 9490059180 | 478708 | 4776950237 | 9857985900 |
| TL-1 | 15305644800 | 14945962147 | 14342919742 | 1311929 | 7280895231 | 14958605400 |
| TL-2 | 14832318000 | 14495624381 | 13918647211 | 1315120 | 6993437937 | 14515924800 |
| TL-3 | 13664940600 | 13346547484 | 12789017908 | 511791 | 6429354552 | 13355162400 |
| TN-1 | 17563924500 | 17114288033 | 16408218268 | 1910554 | 8344620530 | 17262325500 |
| TN-2 | 13469701500 | 13108713500 | 12544333007 | 1433437 | 6361740018 | 13203415200 |
| TN-3 | 14186481600 | 13851680634 | 13319687574 | 1560419 | 6711624445 | 13934348700 |

**Table S3** Overview of the reads and quality control of the 12 libraries of miRNA sequencing from ATII cells

| Sample | Clean_reads | High_quality | Clean_tags |
| --- | --- | --- | --- |
| TL-1 | 13467206 | 13350909 | 11341418 |
| TL-2 | 12806586 | 12699876 | 10781288 |
| TL-3 | 11995365 | 11894344 | 9735117 |
| TN-1 | 11915084 | 11817122 | 9430821 |
| TN-2 | 11761674 | 11587554 | 8191420 |
| TN-3 | 9947404 | 9847843 | 6403578 |
| LL-1 | 10234648 | 10117911 | 6707800 |
| LL-2 | 11285907 | 11211379 | 8984049 |
| LL-3 | 13175380 | 13049934 | 10404999 |
| LN-1 | 11031384 | 10913186 | 8641550 |
| LN-2 | 14071448 | 13925252 | 10772275 |
| LN-3 | 10892875 | 10781661 | 8503588 |
